# Psychosocial factors associated with perceived cognitive functioning in prostate cancer survivors: an exploratory cross-sectional analysis

**DOI:** 10.1007/s00520-025-09771-5

**Published:** 2025-08-02

**Authors:** Lorna Pembroke, Kerry A. Sherman, Haryana M. Dhillon, Heather Francis, David Gillatt, Howard Gurney

**Affiliations:** 1 Lifespan Health and Wellbeing Research Centre, Macquarie University, New South Wales, 2109 Australia; 2 School of Psychological Sciences, Faculty of Medicine, Health and Human Sciences, Macquarie University & Macquarie University Hospital, New South Wales, Macquarie, 2109 Australia; 3 Centre for Medical Psychology and Evidence-Based Decision-Making, School of Psychology, Faculty of Science, University of Sydney, New South Wales, Macquarie, 2006 Australia; 4 Psycho-Oncology Cooperative Research Group, School of Psychology, Faculty of Science, University of Sydney, New South Wales, Macquarie, 2006 Australia; 5 Macquarie University Urology Clinic, Faculty of Medicine and Health Sciences, Macquarie, University & Macquarie University Hospital, New South Wales, Macquarie, 2109 Australia; 6 Macquarie University Clinical Trials Unit (CTU), Faculty of Medicine and Health Sciences, Macquarie University & Macquarie University Hospital, New South Wales, Macquarie, 2109 Australia

**Keywords:** Cognition, Prostate cancer, Psychosocial factors, Quality of life, Hormone therapy

## Abstract

**Purpose:**

As more individuals survive prostate cancer, addressing survivorship concerns like cancer-related cognitive impairment (CRCI) becomes increasingly important. Identifying modifiable psychosocial factors related to CRCI is critical in devising targeted non-pharmacological interventions. We aimed to investigate the psychosocial factors associated with perceived cognitive functioning in prostate cancer survivors (PCS).

**Methods:**

Adult PCS, either undergoing hormone therapy or on ‘watchful waiting’/ ‘active surveillance’, were recruited for a cross-sectional survey. Perceived cognitive functioning was measured using the Perceived Cognitive Impairments subscale (PCI20) from the Functional Assessment of Cancer Therapy- Cognitive Function questionnaire. Pearson’s correlations and ANOVAs explored the association between PCI20 and psychosocial factors including psychological distress, interpersonal functioning, functional well-being, self-compassion and intellectual engagement. Significant variables were included as predictors in a hierarchical regression, examining the relationship with PCI20 and related psychosocial factors while controlling for demographic, biomedical and lifestyle factors.

**Results:**

Of the 96 respondents, one-third reported low cognitive function. Better perceived cognitive functioning was associated with better physical well-being and functional well-being and lower depression levels. In a regression analysis with depression, physical and functional well-being as predictors, only functional well-being was a significant predictor of perceived cognitive functioning after controlling for cancer treatment and levels of physical activity.

**Conclusion:**

Perceived cognitive functioning was associated with self-reported quality of life and the ability to participate in day-to-day activities including work and enjoyment. The use of a biopsychosocial approach in identifying modifiable avenues for therapeutic interventions addressing CRCI may be beneficial.

**Supplementary Information:**

The online version contains supplementary material available at 10.1007/s00520-025-09771-5.

## Introduction

Prostate cancer is one of the most prevalent malignancies, affecting millions worldwide [1]. With advancements in detection and treatment, increasing numbers of individuals are surviving prostate cancer, highlighting the importance of survivorship concerns [2]. Survivorship concerns encompass various factors including quality of life, psychosocial, physical and functional well-being [2]. In particular, cognitive functioning (e.g. memory, attention, planning, problem-solving) is a critical factor in daily functioning, psychosocial well-being, occupational functioning (e.g. return to work), and the management of financial, medical and interpersonal matters [3]. The presence of cancer itself, the experience of a cancer diagnosis and cancer treatments have been associated with cognitive decline, also known as cancer-related cognitive impairment (CRCI) [4, 5]. Though CRCI in non-central nervous system (CNS) cancers is generally mild to moderate, even mild cognitive impairments are associated with reduced self-confidence, greater concerns about changes to interpersonal dynamics and responsibilities, and increased fear of embarrassment [5, 6]. Additionally, there may be more difficulties with decision-making, adhering to treatment and occupational reintegration/performance [7–9].

In prostate cancer, CRCI is complicated by the fact that survivors are more vulnerable to cognitive decline due to older age, with most diagnosed age 65 years or older [10]. The exact mechanisms are not fully understood, but development of CRCI is likely associated with both cancer-related (e.g. cancer stage, treatment, side effects) and non-cancer-related (e.g. demographic, comorbid, genetic, psychosocial) factors [11]. Treatments range from active surveillance to surgery (prostatectomy) to adjuvant therapy, such as hormone (e.g. androgen deprivation), radiation, chemotherapy, immunotherapy or combinations of these. Many treatments have neurotoxic effects or lead to the loss of neuroprotective effects of androgen-related hormones [11]. Moreover, cancer treatments and their side effects may interact with existing risk factors for cognitive impairment (e.g. vascular and metabolic conditions) [12]. Declines in cognitive function have been identified through both neuropsychological testing and self-report measures following common ongoing therapies, such as hormonal treatments, with reported prevalence of CRCI ranging between 10 and 69% [13]. On objective neuropsychological tests declines in processing speed, attention/concentration, verbal/language skills, memory, visuospatial functioning and executive functioning have been documented [14–18]. Moreover, findings from recent systematic review and meta-analysis suggest hormonal treatments increased risk of dementia [19].

Despite comprehensive neuropsychological assessments being the gold standard for assessing cognitive impairment, they are not always feasible to administer in clinical settings. Such assessments are resource intensive, requiring specialised professionals, are financially costly, and often lengthy, taking hours, posing a significant burden, especially for cancer survivors who are likely fatigued and/or cognitively compromised [5, 20]. Self-report measures of cognition provide an efficient, accessible, low-cost option to obtain insights on subjective experiences and the functional impact of CRCI, with an aim to develop tailored interventions [21]. Moreover, decline on self-report measures may be a harbinger of future cognitive decline not yet evident on neuropsychological assessment [22]. Despite weak associations between objective and subjective measures of cognition, perceived cognitive impairment is closely linked with psychological distress, quality of life and day-to-day functioning [23–25].

Given the rapidly growing demand for effective management of CRCI in prostate cancer survivorship, the identification of modifiable factors is a critical initial step in devising targeted interventions as there are no standard treatments [26]. Psychosocial factors have been theorised to contribute to the development and maintenance of perceptions of poor cognitive functioning [27]. Psychosocial factors broadly encompass the cognitive, behavioural, affective and environmental processes or aspects contributing to mental states and social functioning [28]. These factors, unlike many medical, demographic and biological factors, are more readily modifiable and often present important opportunities for intervention in other chronic health conditions [29].

In prostate cancer, depression has been associated with changes in self-reported CRCI [30] and on objective measures [31]. Similarly, in non-CNS cancers more broadly, cognitive complaints have been associated with anxiety and post-traumatic stress symptoms [32, 33]. While continued research is needed to improve understanding about the association between CRCI and psychological distress, limited attention has been given to investigating the relationship between CRCI and other psychosocial factors that may be modifiable targets for intervention in PCS. For instance, in the extant literature on the relationship of psychosocial factors and cognition, evidence suggests the influence of intellectual engagement (i.e. participation in cognitively stimulating/demanding activities), emotional and social support on better cognitive outcomes in later life [34–37]. While use of coping strategies has been associated with self-report cognitive functions in PCS [38], little is known about specific teachable strategies, such as self-compassion (i.e. treating oneself with kindness and understanding and acknowledging and accepting one’s humanity) [39].

Minimal studies have explored the relationship between CRCI and functional well-being in PCS [40], yet they are likely interdependent [35, 41, 42]. Measures of functional well-being can encompass different aspects of a person’s daily functioning and participation, some of which may also be modifiable, such as sleep quality, sense of fulfilment, enjoyability and contentedness [43]. Moreover, examining functioning on an activity/participation level (see the International Classification of Functioning, Disability and Health) [44], not just impairment level, is receiving increasing attention in regard to cognitive rehabilitation for CRCI [45]. Few studies have explored the impact of cognitive rehabilitation on functional outcomes in cancer patients, demonstrating inconsistent benefits [45]. A recent randomised controlled trial, however, found clinically meaningful benefits in daily participation, objective and subjective cognitive functioning following an intervention aiming to improve both cognitive functioning and functional goals in cancer patients with CRCI [46]. Hence, functional well-being is key psychosocial factor to consider when developing interventions aimed at supporting PCS experiencing CRCI.

Our study aimed to investigate associations between psychosocial factors and perceived cognitive functioning in PCS. These factors include psychological distress (i.e. depression and cancer-specific anxiety), interpersonal/social functioning, functional well-being, self-compassion and intellectual engagement.

## Methods

This study reports on the quantitative data of a mixed-methods cross-sectional survey, registered with the Australian New Zealand Clinical Trials Registry on 25 November 2021 (ACTRN12621001608853). Ethics approval was granted by Macquarie University Human Research Ethics Committee (reference number: 52020611919011), in accordance with the Declaration of Helsinki and NHMRC National Statement on Ethical Conduct in Human Research (Commonwealth of Australia, 2007- Updated May 2015). All participants provided written informed consent. Strengthening the Reporting of Observational Studies in Epidemiology (STROBE) checklist was used to guide the reporting of this study [47].

### Participants and procedures

Given the exploratory nature of the study, we recruited PCS, who were literate in English, 18 years and older, either undergoing hormonal treatments (e.g. androgen deprivation therapy) or on ‘watchful waiting’/‘active surveillance’ from Australian healthcare clinics/professionals, HealthMatch, social media (i.e. Facebook, Twitter) and community groups (e.g. Prostate Cancer Foundation Australia support groups, Men’s Sheds, Probus Clubs, etc.) and by passive snowballing between January 2021 to October 2023. After providing consent online, participants were directed to complete self-report questionnaires through online platforms RedCap (Research Electronic Data Capture) [48] and Qualtrics (Provo, UT), or were able to access paper versions in clinic waiting rooms.

### Materials

Demographic, prostate cancer-related and medical information, along with responses to several self-reported questionnaires, were gathered (see Table 1). Self-reported cognitive functioning was measured using the Perceived Cognitive Impairments – 20 items (PCI20) subscale from the Functional Assessment of Cancer Therapy-Cognitive Function (FACT-cog, version 3) questionnaire [49]. Prior research indicates PCI20 yielded correlations with objective cognitive measures, supporting its clinical utility in detecting cognitive impairment [33]. The PCI20 assesses attention, information processing, memory and communication issues (e.g. ‘My thinking has been slow’). Participants rate the frequency of these complaints over the past week on a five-point scale (0 = ‘never’/’not at all’ to 4 = ‘several times a day’/‘very much’), which are reverse coded. Scores range from 0 to 80, with higher scores representing better cognitive function. According to Fardell et al. (2022), scores ≤ 59.5 were indicative of low cognitive function in mixed cancer samples, with 78.8% sensitivity and 84.1% specificity [50]. In our study, Cronbach’s alpha for PCI20 was 0.97.

**Table 1:** Sample demographic, biomedical and lifestyle details and its association with perceived cognitive functioning

	**M (SD),* N* (%), range**	**Association with PCI20 (ANOVA/Kruskal–Wallis/*r*)**	***p*-value**
***Demographic***			
Age (*n* = 96)	*M* = 69.6 (*SD* = 6.9), range = 54.4–84.6	*r* = 0.13	0.22
Relationship (*n* = 96)		*F*(1, 94) = 0.29	0.59
- Partnered	78 (81%)		
- Not partnered	18 (19%)		
Education level (*n* = 96)		*F*(2, 93) = 0.48	0.62
- Did not complete year 12	14 (15%)		
- Year 12/vocational training	28 (29%)		
- Undergraduate/postgraduate	54 (56%)		
Employment status (*n* = 96)		*F*(1, 94) = 0.00	0.96
- Full time/part time/looking for employment/self-employed/carer	33 (34%)		
- Retired	63 (66%)		
Employment type (*n* = 91)		*F*(2, 92) = 1.26	0.29
- Clerical and sales/service/agricultural/machines trade/structural work, e.g. construction	22 (24%)		
- Professional, technical and managerial occupations	56 (62%)		
- Other	13 (14%)		
No. of children (*n* = 83)	*M* = 2.4 (*SD* = 0.9)	*r* = 0.01	0.92
Children living at home (*n* = 84)		*F*(1, 82) = 0.25	0.62
- Yes	11 (13%)		
- No	73 (87%)		
Country of birth (*n* = 95)		*F*(2, 92) = 0.86	0.43
- Aus/NZ	62 (65%)		
- US/UK/Europe	19 (20%)		
- Asia/South America/Africa	14 (15%)		
Location of residence (*n* = 95)		*F*(1, 93) = 0.48	0.49
- Metropolitan	73 (77%)		
- Regional	22 (23%)		
Languages spoken other than English (*n* = 95)		*F*(1, 93) = 0.01	0.90
- No	79 (83%)		
- Yes	16 (17%)		
***Prostate cancer details***			
Stage (*n* = 94)		*χ*^2^(2) = 6.63	0.036*
- Early/localised	46 (49%)		
- Locally advanced	12 (13%)		
- Metastatic	36 (38%)		
Time since diagnosis in years (*n* = 93)	*M* = 5.4 (*SD* = 5.6), range = 0–29	*r* = − 0.056	0.59
Cancer treatments		*χ*^2^(2) = 8.08^a^	0.018*
- Watchful waiting/active surveillance	41 (43%)		
- Surgery	23 (24%)		
- Hormone therapy (e.g. ADT)	55 (57%)		
- Radiation therapy	37 (39%)		
- Chemotherapy	7 (7%)		
- Immunotherapy	3 (3%)		
- Hormone therapy alone	12 (13%)		
- Hormone therapy combined with other treatments	43 (45%)		
Surgery type (*n* = 23)			
- Radical prostatectomy	21 (91%)		
- Other	2 (9%)		
Hormonal treatment (*n* = 55)			
- Goserelin	23 (42%)		
- Leuprolide	15 (27%)		
- Bicalutamide	8 (15%)		
- Enzalutamide	8 (15%)		
- Leuprorelin	7 (13%)		
- Abiraterone	7 (13%)		
- Other	1 (2%)		
Discontinuation of HT due to side effects previously	16 (17%)		
HT administration route (*n* = 49)			
- Monthly injections	4 (8%)		
- Four-monthly injections	31 (63%)		
- Six-monthly injections	5 (10%)		
- Intermittent	3 (6%)		
- Tablets	6 (12%)		
Radiation types (*n* = 35)			
- External beam	32 (91%)		
- Brachytherapy	3 (9%)		
***Medical details***			
Number of medications (*n* = 80)	*M* = 2.9 (*SD* = 2.69), range = 0–15	*r* = −0.13	0.25
No of comorbidities (*n* = 90)	*M* = 0.80 (*SD* = 0.78), range = 0–3	*r* = 0.10	0.35
Heart condition	16 (17%)		
Diabetes	14 (15%)		
High blood pressure	40 (42%)		
Neurological condition	3 (3%)		
Family history of neurological condition	22 (23%)		
Head injury	4 (4%)		
General anesthesia in the past year	39 (41%)		
Stroke history	4 (4%)		
Seizures	2 (2%)		
Mental health	19 (20%)		
***Lifestyle***			
Smoking status (*n* = 94)		*F* (2, 91) = 1.49	0.23
- Never	49 (52%)		
- Currently smoking	3 (3%)		
- Discontinued	42 (45%)		
- > 100 cigarettes in lifetime	30 (32%)		
Alcohol consumption (*n* = 95)		*F*(4, 91) = 0.99	0.42
- I do not drink alcohol	12 (13%)		
- Everyday	20 (21%)		
- A few times a week	34 (36%)		
- A few times a month	13 (14%)		
- A few times a year	16 (17%)		
IPAQ (*n* = 78)		*F*(2, 75) = 3.86	0.025*
- Inactive	17 (22%)		
- Minimally active	27 (35%)		
- HEPA active	34 (44%)		
Diet (*n* = 95)	*M* = 32.3 (*SD* = 4.3), range = 22–40	*r* = − 0.02	0.85

*ADT* = Androgen Deprivation Therapy; *ANOVA*= Analysis of variance; *HEPA* = Health Enhancing Physical Activity; *HT* = Hormonal Treatments; *IPAQ* = International Physical Activity Questionnaire;* PCI20* = FACT-Cog Perceived Cognitive Impairments Subscale – 20 items

a The groups were combined into ‘watchful waiting’/active surveillance, hormone treatments alone, hormone treatments combined with other treatment modalities

* meeting *p <* .05 criteria for inclusion in regression analyses

Psychosocial factors were measured using the following questionnaires, including the physical, functional and social/family well-being subscales of Functional Assessment of Cancer Therapy- Prostate (FACT-P) [43]. Physical well-being was considered a psychosocial factor due to the nature of some scale items tapping into psychosocial functioning (e.g. ‘Because of my physical condition, I have trouble meeting the needs of my family’). Similarly, items of the Functional Well-being subscale addressed cognitive-behavioural processes that may contribute to psychosocial functioning (e.g. ‘I am able to enjoy life’ and ‘I have accepted my illness’). Subjects rated on a five-point scale (from 0 = ‘not at all’ to 4 = ‘very much’) the frequency of various statements over the past week. Scores range from 0 to 28 for each scale. Lower scores indicate poorer quality of life. Cronbach’s alpha was 0.79 for both Physical and Social/Family Well-being scales and 0.78 for the Functional Well-being scale.

Developed and validated in Australia, depression subscale from the short form of Depression Anxiety Stress Scales (DASS-21) [51] was employed as one of the measures of psychological distress. Items assessed symptoms indicative of low mood, lack of interest or pleasure and feelings of worthlessness (e.g. ‘I was unable to become enthusiastic about anything.’). Items were scored on a 4-point Likert scale from 0 = ‘did not apply to me at all’ to 3 = ‘applied to me very much’. Scores range from 0 to 21. Higher scores indicated greater depressive symptoms over the past week. Cronbach alpha was 0.88 in this study.

Cancer-related anxiety was measured using the 4-item Fear of Cancer Recurrence-short form (FCR4) [52]. Each item is rated on a Likert scale ranging from 0 (‘not at all or never’) to 4 (‘a great deal or all the time’). The rating for each item is summed to form a total score. Scores range from 0 to 16. Higher scores indicated greater concerns, fears and worries related to the possibility of cancer progressing or returning (e.g. ‘I get waves of strong feelings about the cancer coming back’). Cronbach’s alpha was 0.95 in this study.

The State Self-Compassion Scale – Short Form [53] contains 6 questions assessing how one responds to painful or difficult situations (e.g. ‘I feel intolerant and impatient toward myself’), measured on a 5- point Likert scale (0 being ‘not at all true for me’ to 4 being ‘very true for me’). Scores range from 0 to 20. Higher scores indicated greater levels of self-compassion. Cronbach’s alpha in this study was 0.63.

Participants’ intellectual engagement was measured using the 6-item version of the Need for Cognition Scale (NCS-6) [54]. Participants rated how each statement applies to them, rated on a Likert scale ranging from 1 (extremely uncharacteristic) to 5 (extremely characteristic). An example of a statement includes ‘I would rather do something that requires little thought than something that is sure to challenge my thinking abilities’. Mean scores range from 1 to 5. Higher mean scores indicated a higher tendency for intellectual engagement. Cronbach’s alpha in this study was 0.77.

Given the potential influence of lifestyle factors on cognitive functions measures of physical activity, diet, smoking status and alcohol consumption were included as potential factors to control for. Smoking status and alcohol consumption levels were captured in the demographics survey. The International Physical Activity Questionnaire (IPAQ) [55], comprised seven items assessing the type and amount of physical activity and time spent sitting over the past 7 days, categorised participants as ‘inactive’, ‘minimally’ active or ‘health-enhancing physical activity’ (HEPA) active. A brief dietary questionnaire, developed by Dr. Heather Francis (author), measured the number of times types of food (e.g. vegetables, fruits) were consumed in a week. Total scores range from 9 to 45. Higher scores indicated greater adherence to the Mediterranean diet. Cronbach’s alpha for the dietary measure in this study was 0.60. Impact of COVID-19 was accounted for using a 3-item questionnaire developed by the research team (see Supplementary Material).

### Statistical analysis

An a priori power analysis conducted using G*Power (version 3.1) for a linear multiple regression with five predictors, *f*^2^ = 0.15, power (1 – *ß*) = 0.80 and *α* = 0.05 indicated a minimum sample size of *N* = 92 [56]. Survey participants, who completed the demographic/medical questions, FACT-P and PCI20, were included for analysis, irrespective of completion status for other questionnaires. Statistical analyses were performed using Statistical Package for the Social Sciences (SPSS, version 29). Missing data were assessed to evaluate the missing at random (MAR) assumption. Missing values ranged from 0.5 to 2% in total within the questionnaires with mean imputation conducted for incomplete responses where possible. Cronbach alphas were calculated for each questionnaire to determine the internal consistency, reliability reported in the measure description.

Scatterplots were initially used to visually examine potential linear or nonlinear associations between perceived cognitive functioning and the demographic/biomedical, psychosocial and lifestyle factors. Based on these observations, Pearson’s correlation analyses were conducted between PCI20 and variables measured on continuous or ordinal scales. Analyses of Variance (ANOVAs) were performed on categorical variables with PCI20 as the dependent variable. Bonferroni-corrected post hoc contrasts were conducted for significant findings. When assumptions of ANOVA were violated, independent-samples Kruskal–Wallis tests were performed, followed by Bonferroni-corrected post hoc comparisons where appropriate. Only variables that were significantly associated (i.e. *p* < 0.05) were included in the regression analyses. A *p* < 0.05 for variable inclusion was adopted to maintain rigor, promote a parsimonious model and limit overfitting given our sample size and study design [57].

Next, a hierarchical regression was performed to examine the relationship between perceived cognitive function and various psychosocial factors after controlling demographic/biomedical and lifestyle factors. In step one of the regression analysis, associated demographic/biomedical and lifestyle factors, identified in earlier analyses, were included to determine the variance explained by these factors. Psychosocial variables were then added in the step two to determine the additional variance explained by these factors and identify significant predictors of perceived cognitive functioning. Standardised residuals were analysed to determine whether there were any outliers. Checks for violations of assumption of collinearity (all tolerance > 0.1, variance inflation factor (VIF) < 10), independent error (Durbin–Watson value = 1.581), normality and homoscedasticity and linearity were also conducted (see Supplementary Material).

## Results

Table 1 contains information regarding sample demographic, biomedical and lifestyle details and its association with perceived cognitive functioning using the PCI20. There were 96 PCS aged 54–85 years (*M* = 69.6, *SD* = 6.9). Most PCS were diagnosed with early/localised (48%) prostate cancer, undergoing watchful waiting/active surveillance (43%) or hormone therapy (e.g. ADT; 13%) and/or with a combination of other treatments (45%). Average time since diagnosis was around 5 years (ranging from less than 1 month to 29 years). A third of participants (32/96 PCS; 33%) reported low cognitive function based off a cutoff score of ≤ 59.5.

### Associations between perceived cognitive impairment and demographic, cancer-related and psychosocial factors

Factors with significant associations (i.e. *p* < 0.05) determined the selection of predictors for the regression analyses.

As outlined in Table 1, there were no significant associations between any demographic or cancer-related factors and scores on the PCI20, except for cancer treatment (*χ*^2^(2) = 8.08, *p* = 0.018), cancer stage (*χ*^2^(2) = 6.63, *p* = 0.036) and physical activity levels (*F*(2,92) = 3.86, *p* = 0.025). Post hoc pairwise comparisons using Bonferroni-adjusted *p*-values indicated that those on watchful waiting/active surveillance (*n* = 41) reported significantly better perceived cognitive functioning (*M* = 68.1, *SD* = 12.0) than those receiving hormonal therapy combined with other treatment modalities (*n* = 43, *M* = 56.9, *SD* = 17.8), *p* = 0.004. There were no significant differences between participants on hormone treatments alone (*n* = 12, *M* = 62.5, *SD* = 17.2) and those undergoing combined treatments (*p* = 0.363) or watchful waiting/active surveillance (*p* = 0.326). Participants with metastatic prostate cancer (*n* = 36, *M* = 56.9, *SD* = 18.7) endorsed significantly worse perceived cognitive functioning than those with early localised/regional prostate cancer (*n* = 58, *M* = 66.2, *SD* = 12.4), *p* = 0.016. No significant differences were observed between the locally advanced and metastatic (*p* = 0.946) or early/localised (*p* = 0.113). Regarding physical activity, participants classified as ‘inactive’ (*n* = 17) reported significantly poorer perceived cognitive functioning (*M* = 53.3, *SD* = 18.6) than those in the HEPA category (*n* = 34, *M* = 65.6, *SD* = 13.5), *p* = 0.026. No significant differences were observed between ‘inactive’ and ‘minimally active’ participants (*p* = 0.169), nor between ‘minimal active’ and HEPA participants (*p* = 1.00).

The associations between cancer treatment, cancer stage and physical activity were also explored. Given the known strong associations between cancer staging and treatment, we conducted a chi-squared test of independence to determine this, which yielded a significant association, *χ*^2^(4) = 53.3, *p* < 0.001. Most participants on ‘watchful waiting’/active surveillance had early or localised prostate cancer (*n* = 37), and those on combined treatments mostly had metastatic (*n* = 26) or locally advanced/regional (*n* = 9) cancer. As cancer treatment was more strongly associated with PCI20 than cancer stage, only cancer treatment was included as a predictor in the regression analysis. The association between cancer treatment and physical activity was also explored using a chi-squared test of independence. Due to expected frequencies below 5 in some cells, Monte Carlo simulation (with 10,000 samples) was implemented finding no significant associations, *χ*^2^(4, *N* = 78) = 4.61, *p* = 0.348.

Regarding the psychosocial factors, higher levels of perceived cognitive functioning were significantly correlated with greater levels of physical well-being (*r* = 0.27, *p* = 0.007), functional well-being (*r* = 0.38, *p* < 0.001), and lower levels of depressive symptoms (*r* =  − 0.22, *p* = 0.035), as reported in Table 2.

**Table 2:** Descriptive data and bivariate correlations between perceived cognitive functioning (PCI20) and psychosocial factors

		**Psychosocial factors**						
	**PCI20**^**a**^	**Physical well-being**^**b**^	**Functional well-being**^**b**^	**Social/family well-being**^**b**^	**Depressive symptoms**	**FCR-4**	**SSCS**	**NCS-6**
***Pearson correlation matrix***								
PCI20 (*n* = 96)	1.00							
Physical well-being (*n* = 96)	0.27**	1.00						
Functional well-being (*n* = 96)	0.38**	0.39**	1.00					
Social/family well-being (*n* = 96)	− 0.01	− 0.12	0.35**	1.00				
Depressive symptoms (*n* = 94)	− 0.22*	− 0.34**	− 0.46**	− 0.19	1.00			
FCR-4 (*n* = 74)	− 0.08	− 0.25*	− 0.29*	− 0.21	0.42**	1.00		
SSCS (*n* = 93)	0.19	0.20	0.35**	0.22*	− 0.64**	− 0.25*	1.00	
NCS-6 (*n* = 95)	0.12	0.11	0.18*	0.05	− 0.12	0.08	0.23*	1.00
***Descriptive data***								
Median	65.1	22.2	21	21	4	4.5	23	3.7
Mean	62.4	21.2	19.8	19.4	4.2	5.4	22.8	3.7
Standard deviation	16.2	5.3	5.3	6.2	3.7	4.3	4.1	0.8
Range	20–80	7–28	6–28	0–31	0–15	0–16	12–30	1.5–5

^a^*PCI20*, perceived cognitive impairments – 20 items subscale from Functional Assessment of Cancer Therapy-Cognitive Subscale; *FCR-4*, 4-Item Fear of Cancer Recurrence-short form; *NCS-6*, 6-item version of Need for Cognition Scale; *SSCS*, State Self-Compassion Scale – Short Form

**p < 0.01

*p < 0.05

### Regression analysis

Table 3 displays the findings of the hierarchical regression analysis. In step 1, both cancer treatment (*β* =  − 0.38, *p* < 0.001) and physical activity (*β* = 0.27, *p* = 0.001) were significant predictors of perceived cognitive functioning, accounting for 21.6% of the variance (*p* ≤ 0.001). When depressive symptoms, physical and functional well-being entered at step 2, the second model explained 29.7% of variance significantly, *F*(5, 72) = 6.09, *p* ≤ 0.001. Along with cancer treatment and physical activity, functional well-being (*β* = 0.30, *p* = 0.014) was also significant predictor of perceived cognitive functioning. This regression had a large effect size (Cohen’s ƒ^2 ^= 0.42).

**Table 3:** Hierarchical linear regression of predictors on perceived cognitive functioning (PCI20)

Step	Variable	*B*	SE (*B*)	*β*	*t*	*R*^2^	*F*	*p*-value
**1**						0.216		
	(Constant)	62.77	3.58		17.54			< 0.001
	Cancer treatment	− 6.46	1.76	− 0.38	− 3.67			< 0.001
	Physical activity	5.39	2.07	0.27	2.60			0.001
**2**						0.297		< 0.001
	(Constant)	62.77	3.57		17.54			0.001
	Cancer treatment	− 5.26	1.91	− 0.31	− 2.76			0.007
	Physical activity	4.46	2.03	0.22	2.20			0.031
	Physical well-being	0.13	0.40	0.04	0.33			0.740
	Functional well-being	0.95	0.38	0.30	2.51			0.014
	Depressive symptoms	0.25	0.53	0.05	0.459			0.648

## Discussion

This study explored associations between various psychosocial factors and perceived cognitive functioning in prostate cancer survivors, which may help inform non-pharmacological interventions to enhance cognitive functioning. We found that a third of PCS reported poorer cognitive function on the PCI20. Of the various psychosocial factors significantly associated with perceived cognitive functioning (i.e. depressive symptoms, physical and functional well-being), only functional well-being predicted perceived cognitive functioning after controlling for cancer treatment and physical activity levels.

The relationship between functional well-being and perceived cognitive functioning may reflect myriad contributing factors associated with prostate cancer and its treatments. While perceived cognitive functioning may be more closely associated with psychological factors than objective cognitive measures [24, 33, 58], physical side effects including pain, sleep disturbances, hot flushes, nausea, genitourinary changes, physical discomfort and fatigue likely disrupt day-to-day functioning and cognition [59]. There may be a bidirectional impact of decline in functioning and cognition [41]. For instance, declines in memory, attention and executive functions (e.g. planning, organising, decision-making) may contribute to difficulties in managing daily tasks and maintaining functional independence. Conversely, declines in day-to-day tasks and mental activities (e.g. financial management, occupational tasks, home maintenance), which commonly occur following PC treatment, may lead to ‘deconditioning’ of cognitive abilities given lack of stimulation [35, 42]. Another consideration includes the close association between self-concept and subjective cognitive functions, whereby poor subjective appraisals of cognitive abilities have been found to be related to decreased confidence in decision-making, ability to attain goals and solve problems [60]. This relationship may explain findings related to functional well-being, assessed by the FACT-P, as items captured an individual’s perceptions of their ability to engage in meaningful activities, perform daily tasks and sense of purpose. Thus, routinely assessing and addressing both CRCI and functional well-being may enhance quality of life in prostate cancer survivorship.

In contrast to previous findings [30], psychological distress was not a significant predictor of cognitive complaints in this sample. Psychological distress was relatively low in our sample, with most participants reporting ‘normal’ to ‘mild’ levels of depressive symptoms. Similarly, the mean response to cancer-specific anxiety was between ‘not at all’ to a ‘little bit’. Response biases may complicate findings, given the tendency for men to underreport psychological symptoms as measures of psychological distress may not sensitively capture symptoms men typically ‘experience’ or recognise [61–63]. Structured clinical interviews and gender-sensitive measures of psychological distress in prostate cancer populations may help mitigate response biases [31]. Nevertheless, assessing psychological distress is clinically important as the coexistence of cognitive complaints and depressive symptoms in older adults may increase the risk of dementia [64].

Though perceived cognitive functioning was not significantly associated with social/family well-being, intellectual engagement and self-compassion, we found these psychosocial factors were associated with functional well-being, suggesting they warrant consideration in the context of enhancing cognitive health. For instance, social support may influence cognitive function through various mechanisms that simultaneously enhance functional well-being. This includes promoting coping strategies, fostering emotional and mental well-being, providing cognitive stimulation through social engagement, encouraging healthy behaviours and advocating for individuals’ healthcare needs [65]. Prior studies have reported frequent engagement in reading, playing musical instruments, playing board games (e.g. chess), attending educational/training courses, cultural engagement and hobbies may reduce the risk of dementia [66–68]. However, our study used a ‘trait’ measure of intellectual engagement, limiting our understanding of how intellectual activities enhance cognitive functions in PCS populations. Moreover, self-compassion interventions have been shown to be an effective behaviour change technique in enhancing self-regulation of health behaviours [69]. Overall, psychosocial factors, including psychological distress, self-compassion, intellectual engagement and social support, should be further explored as avenues for holistic interventions that may promote cognitive function in PCS given its associations with functional well-being.

Our findings were also consistent with existing literature reporting that more advanced cancer and more intensive treatments are often associated with increased cognitive side effects [70]. This may be due to pathophysiological processes including cancer-related inflammation and neurotoxic impacts of treatments, which can disrupt neural functioning [70]. In addition, our findings add to the growing evidence base linking physical activity and cognitive functioning, especially in older populations [71]. Some studies found that physical activity interventions in cancer populations may improve self-reported cognitive function, emotional well-being and fatigue, though effects on objective cognitive measures remain limited [72, 73].

Limitations of our study include the use of self-report measures, which may introduce response biases associated with social desirability, especially when assessing health behaviours and variability in memory recall abilities, level of self-awareness and insight. Shared method variance (i.e. subjective measures are self-reported) may conflate the association between psychosocial and cognitive functioning. Moreover, self-selection bias can obscure the generalisability of the findings as most participants in this study were highly educated, suggesting higher levels of cognitive reserve (i.e. ability to withstand injury to the brain) [74], thus lower susceptibility to CRCI. Study samples with lower education (e.g. mean of 10–11 years) demonstrated more observable cognitive impairments than samples with higher education levels (e.g. median of 16 years) following hormonal treatments for prostate cancer [15, 18, 75]. Nonlinear relationships between the study variables were not statistically evaluated and warrant consideration in future studies. Additionally, the study was not adequately powered to examine the association between specific cognitive domains and various psychosocial factors. Furthermore, given the limitations of the cross-sectional design, prospective longitudinal research is required to investigate the directional relationship between perceived cognitive functions and psychosocial factors and their impact on day-to-day functioning.

In conclusion, there is a growing need to addressing CRCI in prostate cancer survivorship. Our findings highlight the association between CRCI and functional well-being in PCS, which should be considered in the development of therapeutic interventions for CRCI. Future research should use biopsychosocial and multidisciplinary approaches to identify at-risk individuals and inform targeted treatments, promoting a comprehensive understanding of CRCI to enhance survivorship care.

## Supplementary Information

Below is the link to the electronic supplementary material.

Supplementary file1 (DOCX 446 KB)

## Data Availability

Data and materials may be made available upon request.
