# Supplementary material for: Psychosocial factors associated with perceived cognitive functioning in prostate cancer survivors: an exploratory cross-sectional analysis
Source: Support Care Cancer. 2025 Aug 2;33(8):744. doi: 10.1007/s00520-025-09771-5 (PMC12317862; doi:10.1007/s00520-025-09771-5)

[COVID-19 impact questions 2](#_Toc168398658)

[Assumptions of Regression Analyses 3](#_Toc168398659)

## COVID-19 impact questions

For the following questions, please select the number that best corresponds to your views.

1. How much is COVID-19 affecting your life?

0 (no affect at all) -> 10 (severely affects my life)

1. How concerned are you about COVID-19

0 (not at all concerned) -> 10 (extremely concerned)

1. How much does COVID-19 affect you emotionally (e.g., does it make you angry, scared, upset or depressed?)

0 (not at all affected emotionally) -> 10 (extremely affected emotionally)

## Assumptions of Regression Analyses

Checks for violations of assumption of collinearity (all tolerance > 0.1, variance inflation factor (VIF) < 10), independent error (Durbin–Watson value = 1.374), normality and homoscedascity and linearity were also conducted.


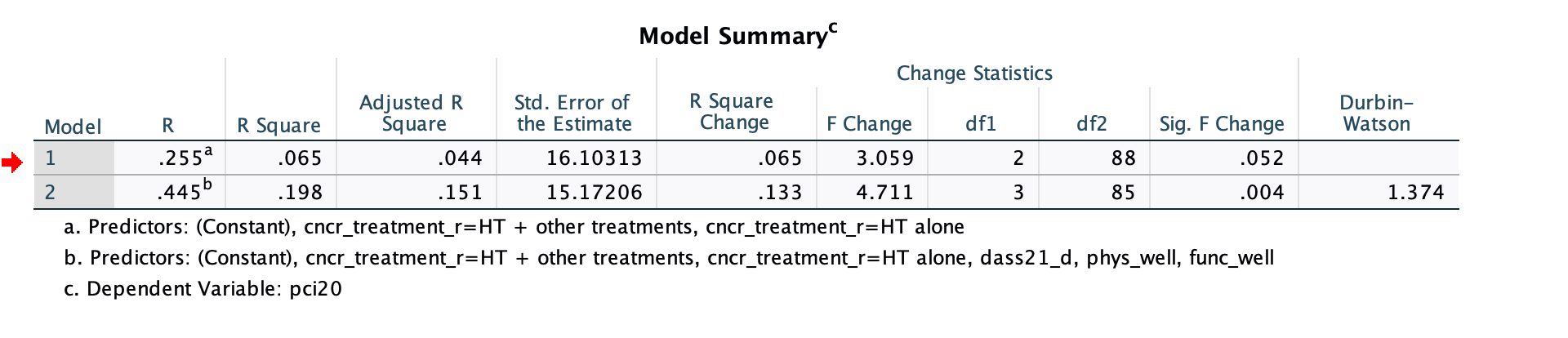


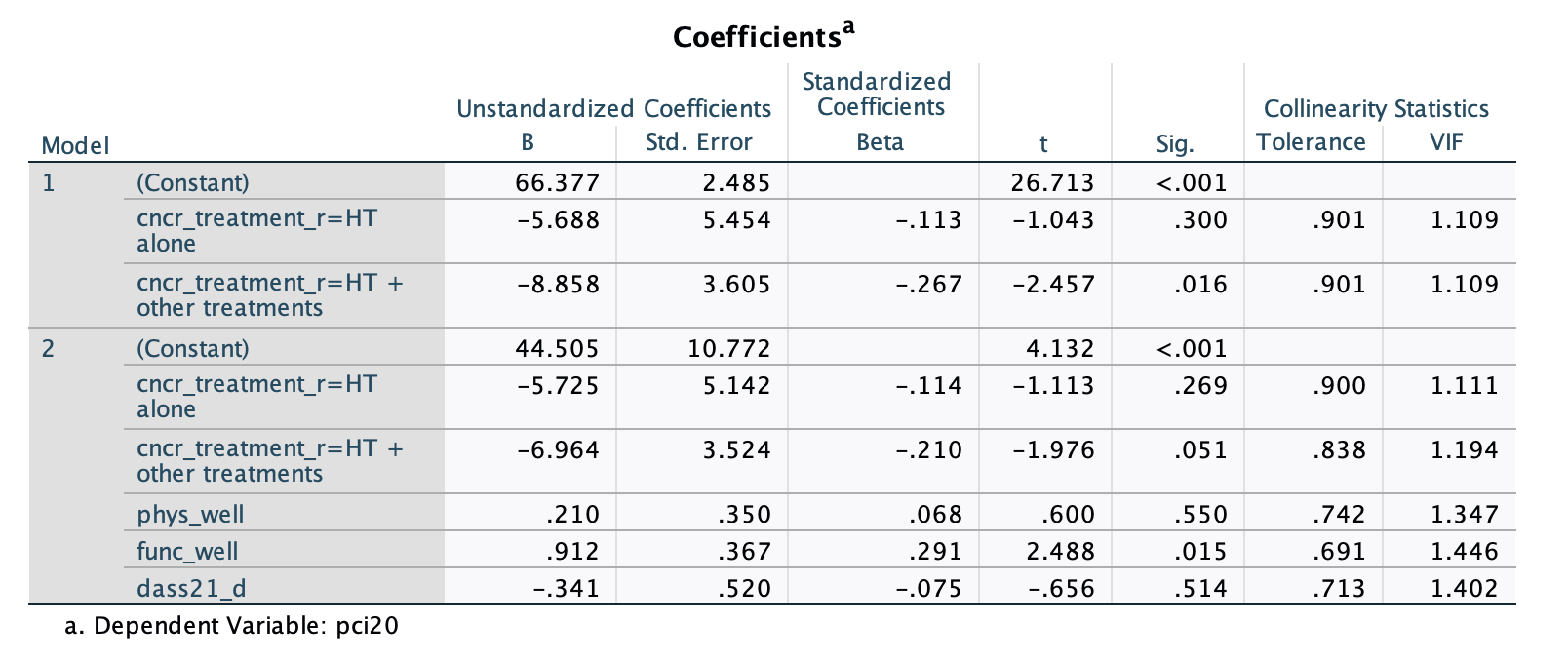


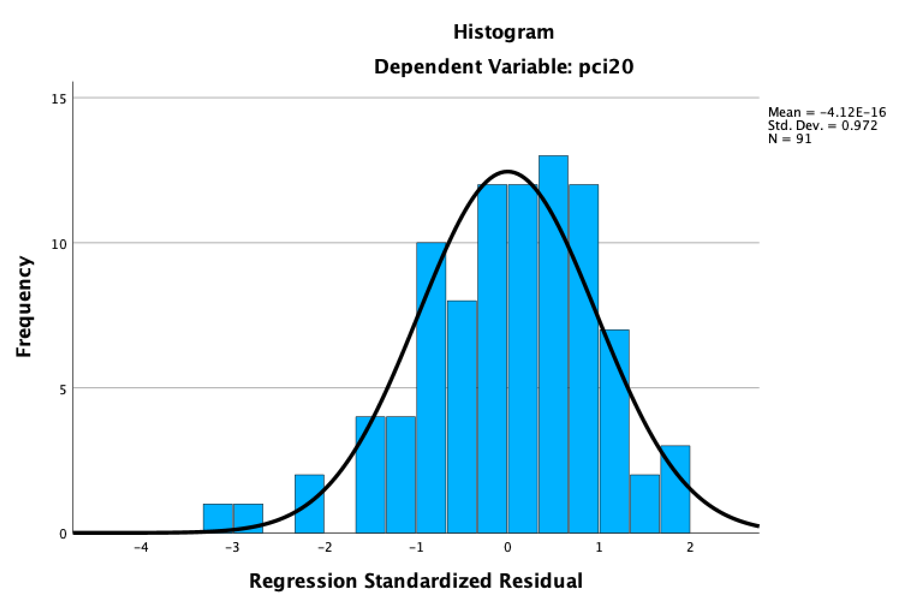

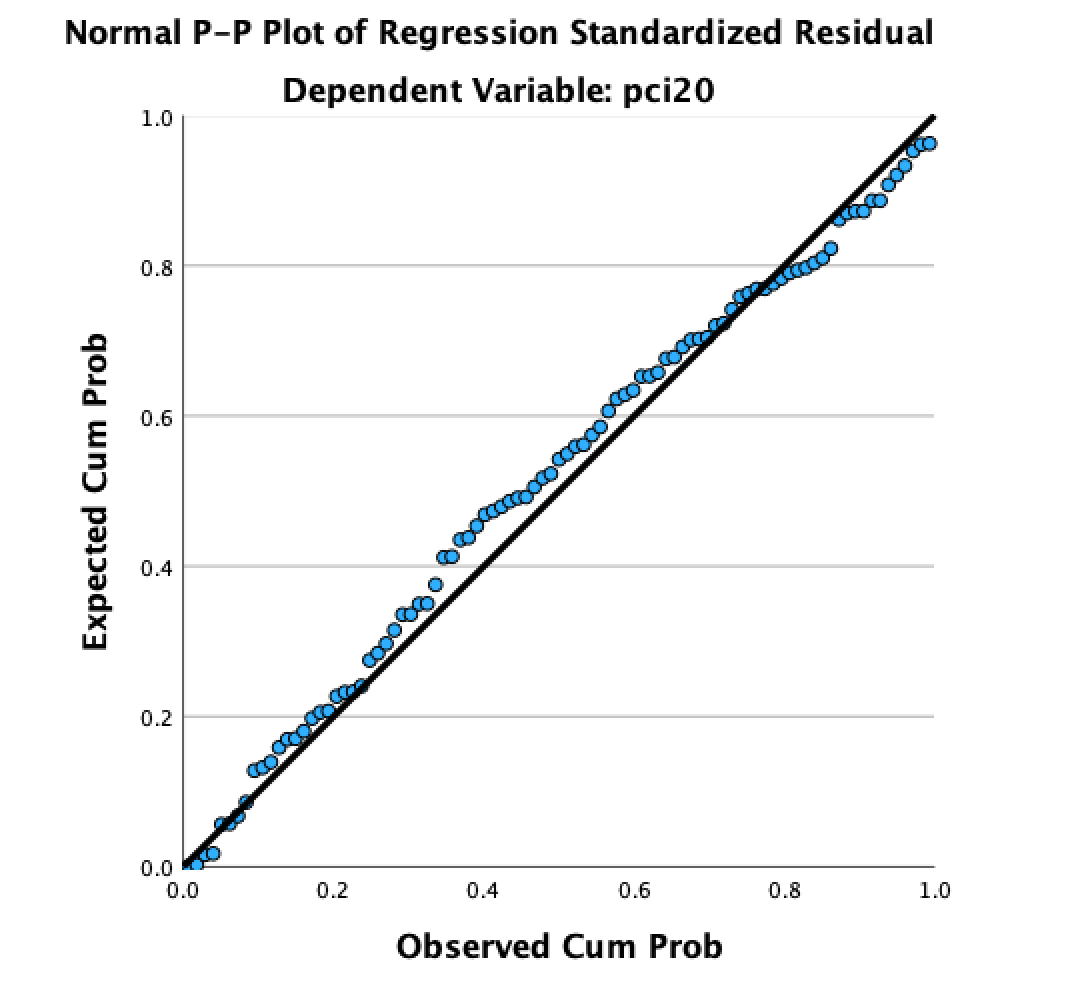


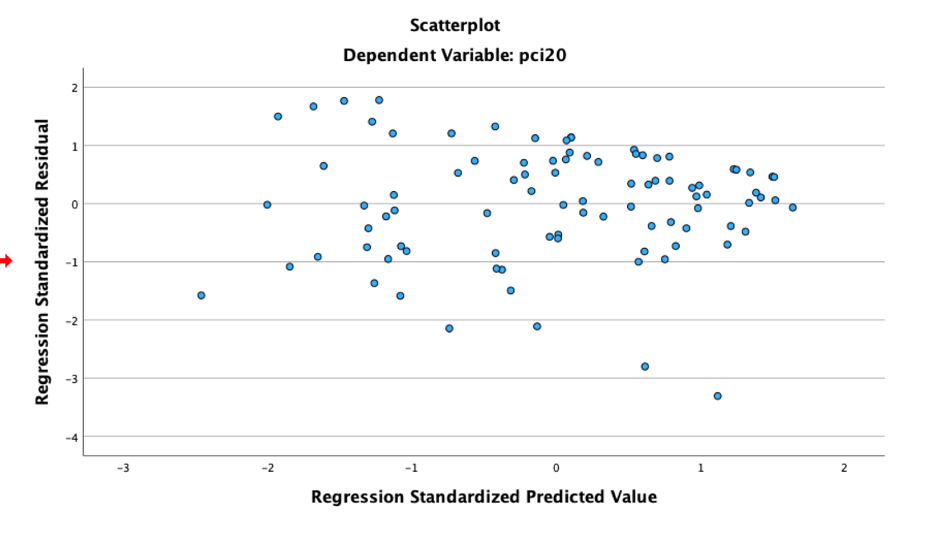

Supplement: Supplementary file 1 — Supplementary file1 (DOCX 446 KB) [file 520_2025_9771_MOESM1_ESM.docx]
